# Relationships between disaccharidase deficiencies, duodenal inflammation and symptom profile in children with abdominal pain

**DOI:** 10.1038/s41598-021-84535-9

**Published:** 2021-03-01

**Authors:** Jennifer M. Colombo, Chance S. Friesen, Uttam Garg, Craig A. Friesen, William San Pablo

**Affiliations:** 1 Division of Gastroenterology, Hepatology, and Nutrition, Children’s Mercy Hospital, 2401 Gillham Road, Kansas City, MO 64109 USA; 2 Pathology and Laboratory Medicine, Children’s Mercy Hospital, 2401 Gillham Road, Kansas City, MO 64108 USA

**Keywords:** Gastroenterology, Pathogenesis

## Abstract

Abdominal pain has been associated with disaccharidase deficiencies. While relationships with individual symptoms have been assessed, relationships between disaccharidase deficiencies and symptom complexes or inflammation have not been evaluated in this group. The primary aims of the current study were to assess relationships between disaccharidase deficiency and symptoms or symptom complexes and duodenal inflammation, respectively. Patients with abdominal pain who underwent endoscopy with evaluation of disaccharidase activity levels were identified. After excluding all patients with inflammatory bowel disease, celiac disease, *H. pylori*, or gross endoscopic lesions, patients were evaluated for disaccharidase deficiency frequency. Disaccharidase were compared between patients with and without histologic duodenitis. Lastly, relationships between individual gastrointestinal symptoms or symptom complexes were evaluated. Lactase deficiency was found in 34.3% of patients and disaccharidase pan-deficiency in 7.6%. No individual symptoms or symptom complexes predicted disaccharidase deficiency. While duodenitis was not associated with disaccharidase deficiency, it was only present in 5.9% of patients. Disaccharidase deficiency, particularly lactase deficiency, is common in youth with abdominal pain and multiple deficiencies are not uncommon. Disaccharidase deficiency cannot be predicted by symptoms in this population. Further studies are needed to assess the clinical significance of disaccharidase deficiency.

## Introduction

Chronic abdominal pain is common in children and adolescents affecting up to 19–38% of the pediatric population^1, 2^. Most of these children will fulfill criteria for an abdominal pain-related functional gastrointestinal disorder as defined by Rome IV criteria with the two most common being irritable bowel syndrome (IBS) and functional dyspepsia (FD)^3–6^. IBS is defined as pain in association with a change in stool frequency, a change in stool form, or a change in pain with defecation^4^. FD is defined by epigastric pain or burning, early satiety, or postprandial bloating^4^.

Disaccharidase (DS) deficiencies have also been implicated in children with chronic abdominal pain^7–9^. These may be primary deficiencies in patients with normal duodenal histology and presumably genetic in origin or may be secondary to mucosal injury with villous atrophy and/or inflammation. With DS deficiency, ingestion of the corresponding disaccharide can result in malabsorption with subsequent osmotic diarrhea, cramping, and increased gas with bloating following bacterial digestion of the sugar^10^. Despite these physiologic and symptom consequences, previous studies have not been able to correlate DS deficiencies with specific symptoms in youth with abdominal pain^7, 8^.

The primary aims of the current study were to, (1) assess the frequency of DS deficiency in children and adolescents with chronic abdominal pain, (2) to assess relationships between DS deficiencies and specific symptoms or groups of symptoms corresponding to symptoms generally associated with DS deficiency, IBS, and FD, respectively, and, (3) to assess relationships between duodenal inflammation and DS activity and deficiencies. In addition, we evaluated whether reports of specific food intolerances correlated with DS deficiencies. We hypothesized that DS deficiency would be common in youth with chronic abdominal pain but would not be associated with specific symptoms or symptom complexes.

## Methods

This study was a retrospective, single-site study of all children and adolescents evaluated in an abdominal pain clinic who had undergone endoscopy for a primary indication of abdominal pain who also had evaluation of duodenal disaccharidase (DS) activity at Children’s Mercy Kansas City from 2016 to 2019. The abdominal pain clinic evaluates patients 8–17 years of age with a pain duration of at least 8 weeks. Electronic medical records were reviewed and patients were excluded for a previous diagnosis of inflammatory bowel disease or celiac disease. All diagnoses had been assigned by a board-certified pediatric gastroenterologist based on clinical, laboratory, and histologic findings. All patients with celiac disease had an elevated tissue transglutaminase antibody level. Patients’ medical records were also reviewed for gross endoscopic findings and histologic findings including identification of *Helicobacter pylori*. Patients with *H. pylori* on histology as determined by a board-certified pediatric pathologist and patients with endoscopic findings of erosions, ulcers, or nodular gastritis were also excluded. Microscopic inflammation did not otherwise preclude inclusion. When present inflammation within duodenum was further classified as chronic (increased lymphocytes or plasma cells), acute (neutrophils alone or in addition to chronic inflammation), or eosinophilic (increased eosinophils alone or in addition to chronic inflammation). All histologic evaluations for inflammation and villous atrophy had been performed as part of routine care by board certified pediatric pathologists. All patients within the abdominal pain clinic undergo a standardized comprehensive history for the presence of gastrointestinal symptoms. Gastrointestinal symptoms are captured electronically and patients are required to answer the complete set of symptom questions. In addition, all patients are asked if there are specific foods or types of foods which are associated with increased pain. Patients’ medical records were utilized to assess relationships between DS deficiencies and symptoms and reported food triggers. All DS activity analyses were performed in a commercial clinical laboratory (Joli Diagnostics, Inc.) utilizing the modified Dahlqvist method to assess enzyme activity levels. Both actual DS activity levels and whether levels were normal or abnormal per reference laboratory normal ranges were recorded and analyzed. Normal values include lactase > 15 μmol/min/g protein, sucrose > 25 μmol/min/g protein, maltase > 100 μmol/min/g protein, and palatinase > 5 μmol/min/g protein. This study was approved by the Institutional Review Board Children’s Mercy Kansas City which also waived informed consent and assent for this retrospective study. All research was performed in accordance with relevant guidelines/regulations.

### Statistical analysis

Statistical analyses were performed using SPSS version 23 (SPSS Inc., Chicago, IL). The frequencies of DS deficiencies were compared between those with and without specific individual symptoms by chi square analysis. The ability of three separate sets of symptoms designated set 1 (loose stools, bloating, and increased gas), set 2 (upper abdominal pain, postprandial bloating, and early satiety), and set 3 (change in stool form, change in stool frequency, and pain relief with a stool) to predict DS deficiency were assessed by logistic regression. Frequencies of DS deficiency were compared between patients with duodenitis and those without duodenitis by chi square analysis. The frequencies of DS deficiencies in patients reporting an increase in pain with dairy ingestion as compared to those not reporting an increase in pain were compared by chi square analysis. A p value < 0.05 was considered significant.

## Results

Patients ranged in age from 8 to 17 years with a mean age of 13.4 ± 2.9 years and 72.4% were female. The pain duration ranged from 2 to 204 months with a mean of 33.2 ± 35.7 months. The pain was continuous in 28.8%, daily intermittent in 40.5%, several days/week in 20.7%, and weekly in 9.9%.

Lactase deficiency was present in 34.3%, sucrase deficiency in 7.6%, maltase deficiency in 10.9%, palatinase deficiency in 7.6%, and pan-deficiency in 7.6%. The prevalence of DS deficiencies did not differ between patients with and without duodenitis, however, duodenitis was only present in 5.9% of patients (one chronic, one acute, and five eosinophilic).

Frequency of gastrointestinal symptoms are shown in Table 1. No single symptom was associated with an increased frequency of deficiency for any DS. Logistic regression was performed for three symptom sets (corresponding to DS deficiency, FD, and IBS) evaluating their ability to predict the presence of any specific DS deficiency. There were no significant associations between any symptom set and lactase, sucrase, maltase, or palatinase deficiency.

**Table 1:** Symptom frequencies in APP patients evaluated in the abdominal pain program.

Nausea: 82.4%
Increased pain with eating: 69.7%
Periumbilical pain: 66.4%
Upper pain: 62.2%
Early satiety: 58.8%
Postprandial bloating: 52.9%
Lower pain: 48.7%
Excess gas: 31.9%
Pain improved with stool: 31.1%
Change is stool form: 30.3%
Change in stool frequency: 29.4%
Diarrhea/loose stools: 29.4%
Vomiting: 24.4%
Weight loss: 11.8%

For patients reporting increased pain with eating, a specific food or food type which increased pain was identified by 61.4%. The most common were dairy in 28.9%, fried or greasy foods in 20.5%, spicy foods in 13.3%, sugar or sweets in 6.0%, and gluten or bread in 4.8%. Lactase deficiency alone was seen at an increased frequency in patients reporting increased pain with dairy consumption (Table 2). Only one of the five patients reporting increased pain with sugar or sweets had a sucrase deficiency.

**Table 2:** Frequencies of disaccharidase deficiencies in abdominal pain program patients (for whom eating increases pain) reporting increased pain with dairy intake as compared to those not reporting increased pain with dairy intake.

	Dairy intolerance (N = 24) (%)	No dairy intolerance (N = 27) (%)	p value
Lactase	62.5	25.9	0.008
Sucrase	8.3	3.7	0.483
Maltase	16.7	3.7	0.120
Palatinase	8.3	3.7	0.483

## Discussion

DS deficiency, particularly lactase deficiency, is common in children and adolescents with chronic abdominal pain, even in patients with normal duodenal histology. The low prevalence of duodenitis in the current study precluded meaningful evaluation of any association of duodenitis and DS deficiency. While normal histology may indicate a primary or genetic deficiency, it is also possible that the DS activity levels were subject to alteration due to microbiome composition or differences in dietary intake as suggested by animal models^11, 12^. Chumpitazi et al. found lactase deficiency in nearly half of youth with functional dyspepsia with an additional DS deficiency in 7.9–17.9%^7^. Another study of children with dyspepsia with normal histology reported DS deficiency in 50%^9^. El-Chammas et al. studied children with chronic abdominal pain and normal histology and found lactase deficiency in 37% and sucrase deficiency in 21%^8^. We found a pan-deficiency in 7.3% which has also been reported by others where pan-deficiency was not uncommon^7^. It has been suggested that apparent pan-deficiency may be an artifact due to improper acquisition of biopsies (e.g. from the duodenal bulb) or inappropriate processing of the sample (e.g. delay in freezing of the sample). While this is possible in some cases, pan-deficiency is also seen frequently even with meticulous biopsy procedures and immediate processing of the sample^7^. Pan-deficiency may also result from a combined deficiency of lactase, which is common, and sucrase-isomaltase which accounts for sucrase and palatinase activity as well as 80% of maltase activity^13^.

Similar with previous studies, we found that no individual symptoms predicted the presence of DS deficiency^7, 8^. In addition, we found that symptom complexes consistent with either IBS or FD did not improve this predictive ability, nor did a complex of symptoms which have generally been associated with DS deficiency (loose stools, bloating, and increased gas). Previous studies have reported some associations between congenital sucrase-isomaltase deficiencies and IBS but no clear association of lactase deficiency with IBS^14–16^. A previous study utilizing the 13C-sucrose breath test in children found no association between sucrase deficiency and specific symptoms [^17^]. The lack of association between specific symptoms and DS deficiency in patients with chronic abdominal pain should not be surprising as there are a variety of factors which affect symptoms directly related to disaccharide ingestion as well as a variety of factors affecting symptom which may not be directly related such as dysmotility, visceral hyperalgesia, and psychosocial functioning. In addition, the frequency or severity of symptoms related to disaccharides are dependent on the degree of enzyme deficiency, the amount ingested, other components in the diet, and gastrointestinal motility^18^. A recent study in mice provided a possible link between disaccharide malabsorption and visceral hyperalgesia as oral administration of foods containing fructo-oligosaccharide or lactose gavage led to increased abdominal sensation in association with an increase in colonic mast cells^19^. Whether DS deficiency and malabsorption is a contributor to the development of visceral hyperalgesia in humans remains to be determined but it represents an important area of inquiry.

While the clinical significance of identified DS deficiency requires further evaluation, the practice of obtaining mucosal specimens for DS analysis frequently leads to a change in therapy, either dietary restriction or enzyme replacement. This is particularly true of patients with lactase deficiency where changes in treatment were recommended in 85% of patients. In the current study, perceived dairy intolerance was the most common reported food resulting in increased pain. In these patients there was an increased frequency of lactase deficiency but not other DS deficiencies suggesting that milk intolerance may be related to lactose malabsorption in at least some patients. The higher reporting of dairy intolerance in the current study is consistent with previous studies^20,21^. Despite this, there is a lack of compelling evidence that restriction of individual disaccharides improves symptoms^18^. Specifically, randomized studies assessing lactose restriction have produced mixed results but generally do not support significant benefit^18^.

The strengths of the current study include inclusion of a group of patients with prospectively collected standardized and comprehensive gastrointestinal symptom histories with analysis of DS relationships to sets of symptoms. The primary weakness is that DS analysis is not universally performed with endoscopy and thus the patients are pre-selected based on the clinical judgement of the ordering clinician.

In conclusion, DS deficiency occurs commonly in youth with abdominal pain and normal histology. Neither individual gastrointestinal symptoms nor complexes of symptoms corresponding to “usual” DS malabsorption symptoms, IBS, or FD were predictive of DS deficiency. While an increased frequency of lactase deficiency in patients reporting increased pain with dairy would suggest a relationship to symptoms, well-designed prospective studies are needed to assess the efficacy of disaccharide restriction, either individually or as a group, in improving pain and whether DS activity levels are predictive of response.

## Abbreviations

DS: Disaccharidase
IBS: Irritable bowel syndrome
FD: Functional dyspepsia
APP: Abdominal pain program
